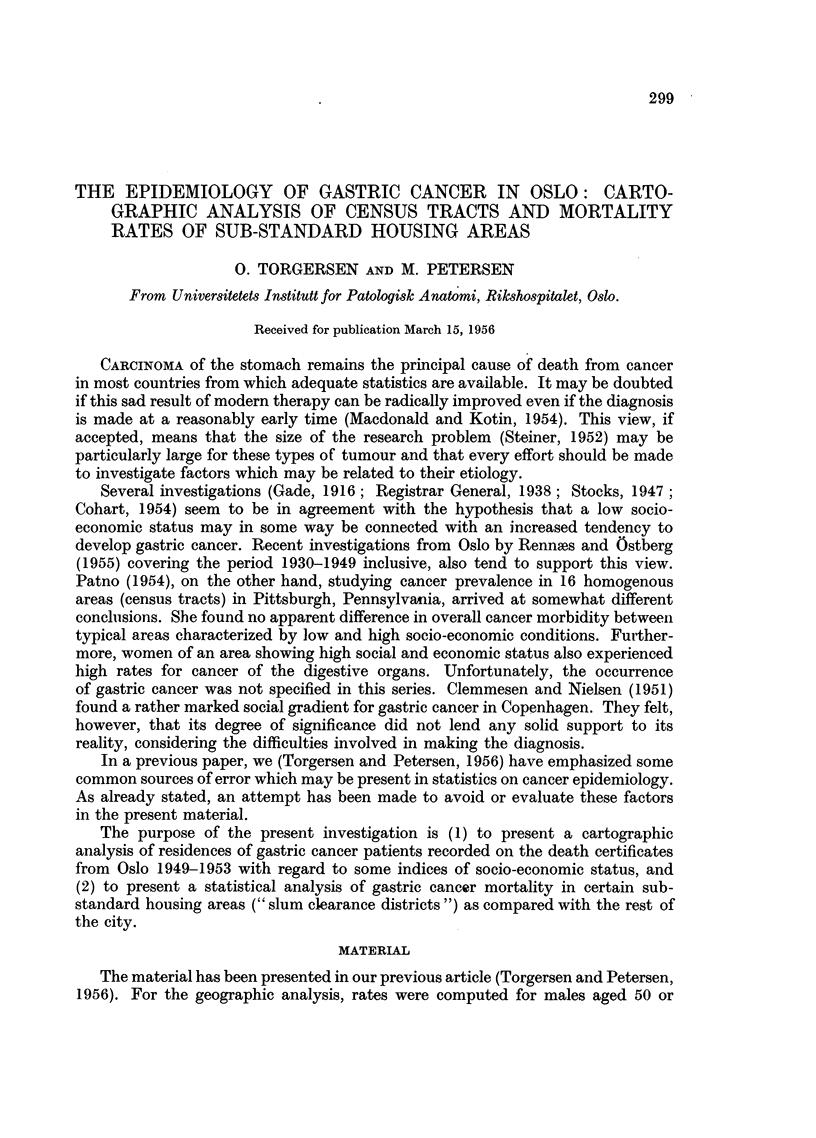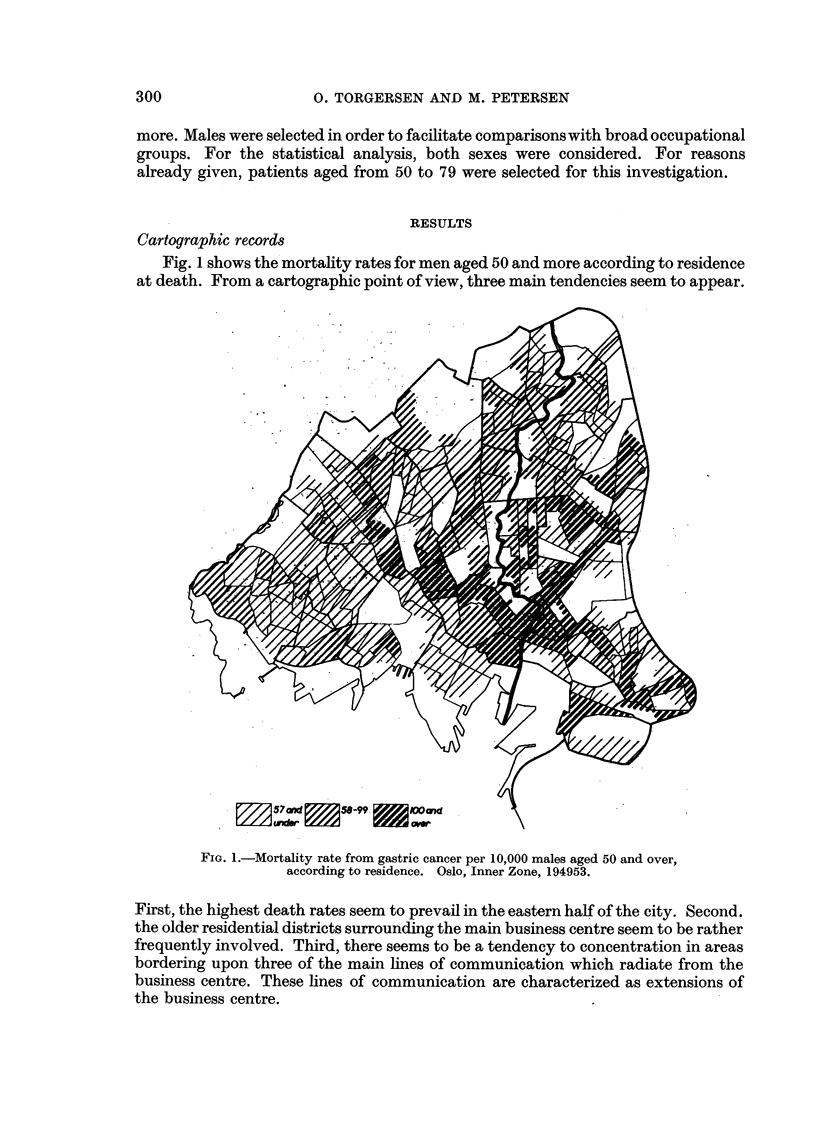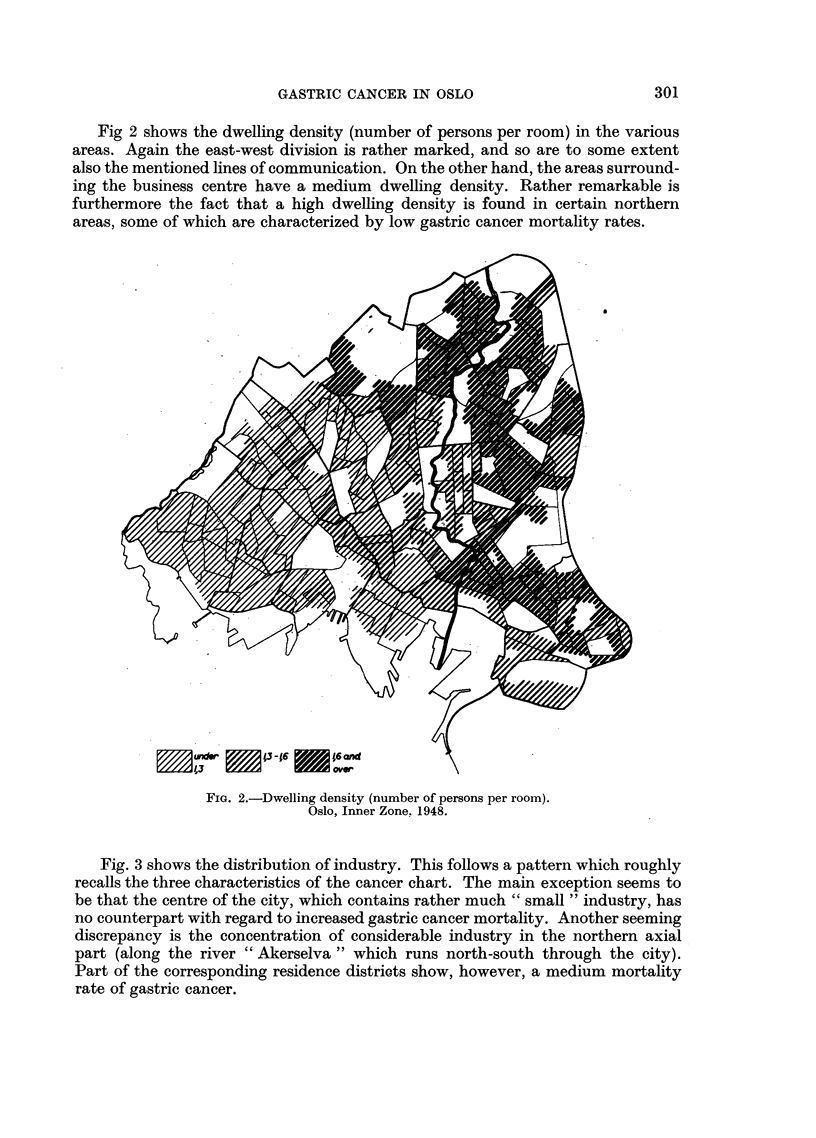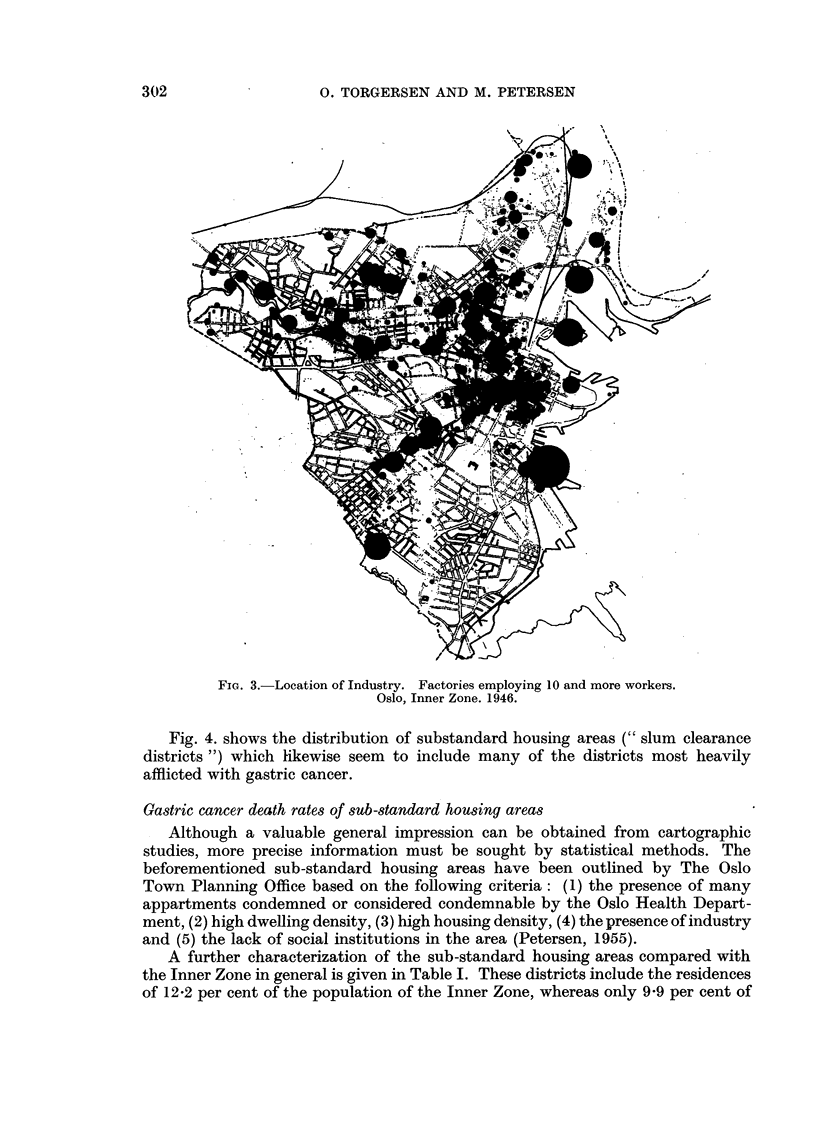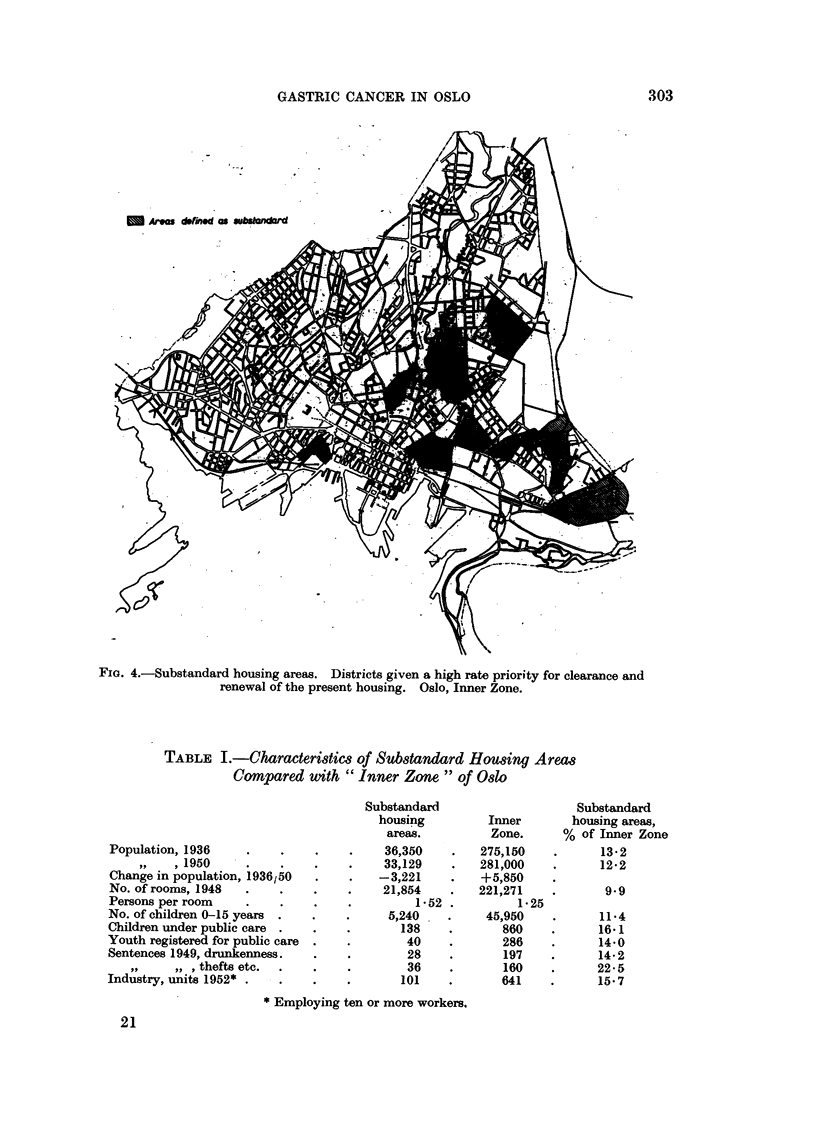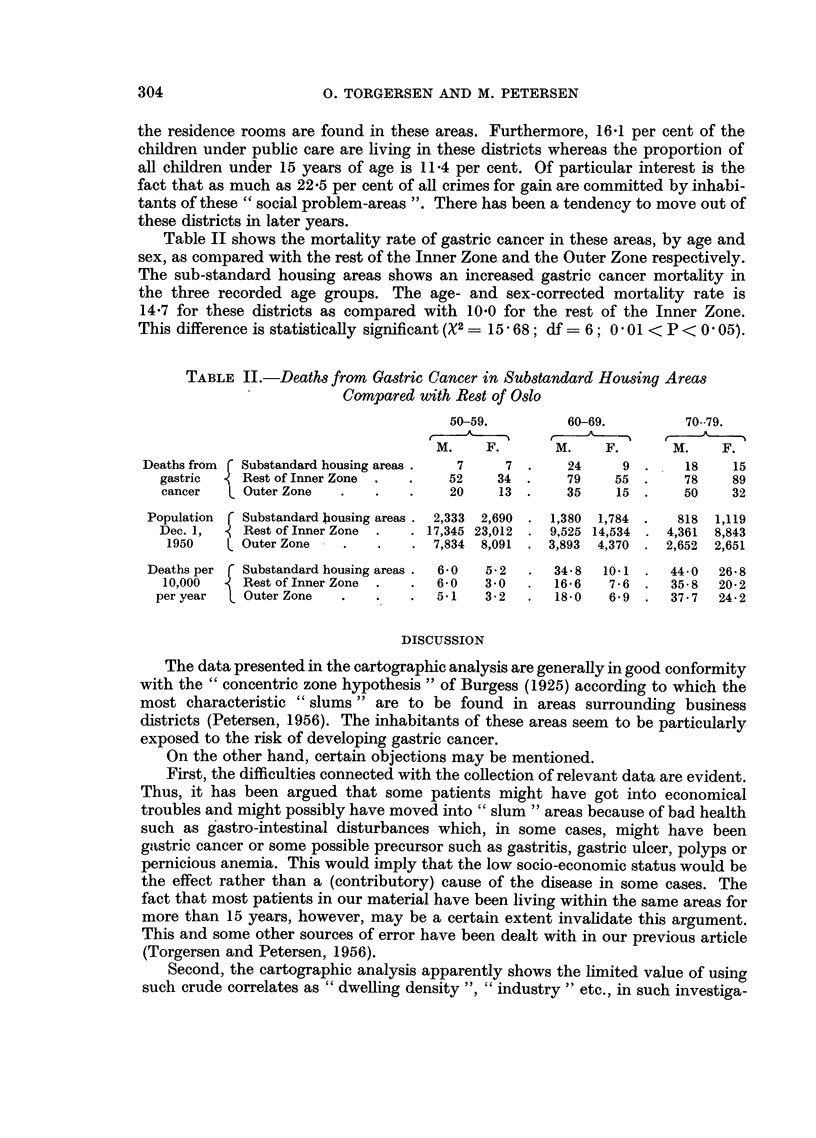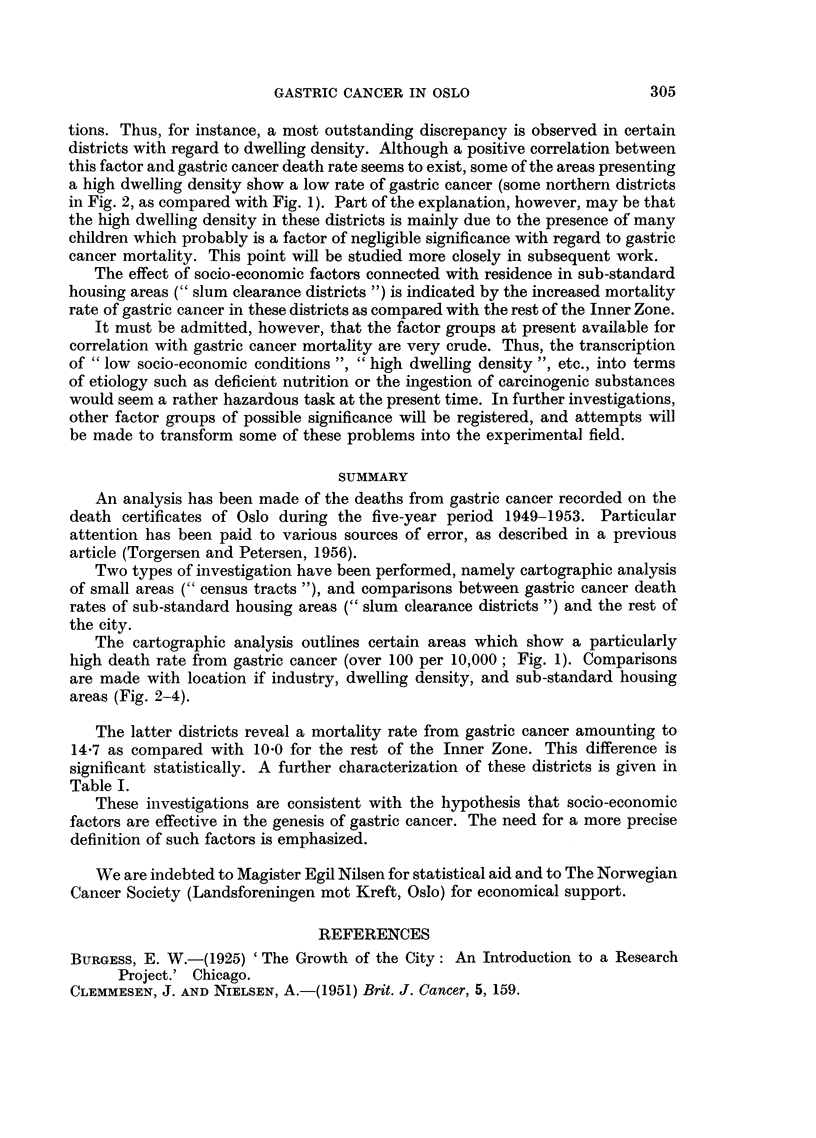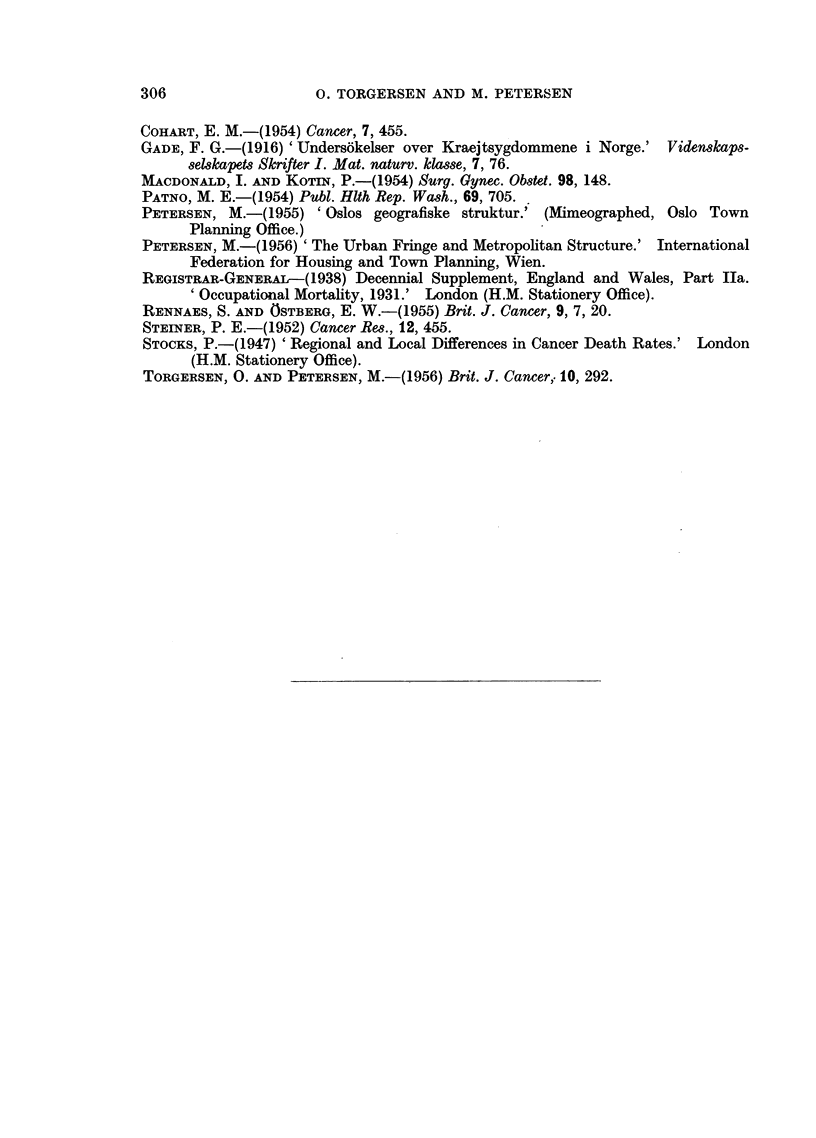# The Epidemiology of Gastric Cancer in Oslo: Cartographic Analysis of Census Tracts and Mortality Rates of Sub-Standard Housing Areas

**DOI:** 10.1038/bjc.1956.34

**Published:** 1956-06

**Authors:** O. Torgersen, M. Petersen


### THE EPIDEMIOLOGY OF GASTRIC CANCER IN OSLO: CARTOGRAPHIC ANALYSIS OF CENSUS TRACTS AND MORTALITY RATES OF SUB-STANDARD HOUSING AREAS

O. TORGERSEN AND M. PETERSEN

*From Universitetets Institutt for Patologisk Anatomi, Rikshospitalet, Oslo.*

Received for publication March 15, 1956

CARCINOMA of the stomach remains the principal cause of death from cancer in most countries from which adequate statistics are available. It may be doubted if this sad result of modern therapy can be radically improved even if the diagnosis is made at a reasonably early time (Macdonald and Kotin, 1954). This view, if accepted, means that the size of the research problem (Steiner, 1952) may be particularly large for these types of tumour and that every effort should be made to investigate factors which may be related to their etiology.

Several investigations (Gade, 1916; Registrar General, 1938; Stocks, 1947; Cohart, 1954) seem to be in agreement with the hypothesis that a low socio-economic status may in some way be connected with an increased tendency to develop gastric cancer. Recent investigations from Oslo by Rennæs and Östberg (1955) covering the period 1930–1949 inclusive, also tend to support this view. Patno (1954), on the other hand, studying cancer prevalence in 16 homogenous areas (census tracts) in Pittsburgh, Pennsylvania, arrived at somewhat different conclusions. She found no apparent difference in overall cancer morbidity between typical areas characterized by low and high socio-economic conditions. Furthermore, women of an area showing high social and economic status also experienced high rates for cancer of the digestive organs. Unfortunately, the occurrence of gastric cancer was not specified in this series. Clemmesen and Nielsen (1951) found a rather marked social gradient for gastric cancer in Copenhagen. They felt, however, that its degree of significance did not lend any solid support to its reality, considering the difficulties involved in making the diagnosis.

In a previous paper, we (Torgersen and Petersen, 1956) have emphasized some common sources of error which may be present in statistics on cancer epidemiology. As already stated, an attempt has been made to avoid or evaluate these factors in the present material.

The purpose of the present investigation is (1) to present a cartographic analysis of residences of gastric cancer patients recorded on the death certificates from Oslo 1949–1953 with regard to some indices of socio-economic status, and (2) to present a statistical analysis of gastric cancer mortality in certain sub-standard housing areas ("slum clearance districts") as compared with the rest of the city.

### MATERIAL

The material has been presented in our previous article (Torgersen and Petersen, 1956). For the geographic analysis, rates were computed for males aged 50 or more. Males were selected in order to facilitate comparisons with broad occupational groups. For the statistical analysis, both sexes were considered. For reasons already given, patients aged from 50 to 79 were selected for this investigation.

### RESULTS

### *Cartographic records*

Fig. 1 shows the mortality rates for men aged 50 and more according to residence at death. From a cartographic point of view, three main tendencies seem to appear.

FIG. 1.—Mortality rate from gastric cancer per 10,000 males aged 50 and over, according to residence. Oslo, Inner Zone, 194953.

First, the highest death rates seem to prevail in the eastern half of the city. Second. the older residential districts surrounding the main business centre seem to be rather frequently involved. Third, there seems to be a tendency to concentration in areas bordering upon three of the main lines of communication which radiate from the business centre. These lines of communication are characterized as extensions of the business centre.

Fig 2 shows the dwelling density (number of persons per room) in the various areas. Again the east-west division is rather marked, and so are to some extent also the mentioned lines of communication. On the other hand, the areas surrounding the business centre have a medium dwelling density. Rather remarkable is furthermore the fact that a high dwelling density is found in certain northern areas, some of which are characterized by low gastric cancer mortality rates.

FIG. 2.—Dwelling density (number of persons per room). Oslo, Inner Zone, 1948.

Fig. 3 shows the distribution of industry. This follows a pattern which roughly recalls the three characteristics of the cancer chart. The main exception seems to be that the centre of the city, which contains rather much " small " industry, has no counterpart with regard to increased gastric cancer mortality. Another seeming discrepancy is the concentration of considerable industry in the northern axial part (along the river " Akerselva " which runs north-south through the city). Part of the corresponding residence districts show, however, a medium mortality rate of gastric cancer.

FIG. 3.—Location of Industry. Factories employing 10 and more workers.
Oslo, Inner Zone. 1946.

Fig. 4. shows the distribution of substandard housing areas (" slum clearance districts ") which likewise seem to include many of the districts most heavily afflicted with gastric cancer.

*Gastric cancer death rates of sub-standard housing areas*

Although a valuable general impression can be obtained from cartographic studies, more precise information must be sought by statistical methods. The beforementioned sub-standard housing areas have been outlined by The Oslo Town Planning Office based on the following criteria : (1) the presence of many appartments condemned or considered condemnable by the Oslo Health Department, (2) high dwelling density, (3) high housing density, (4) the presence of industry and (5) the lack of social institutions in the area (Petersen, 1955).

A further characterization of the sub-standard housing areas compared with the Inner Zone in general is given in Table I. These districts include the residences of 12·2 per cent of the population of the Inner Zone, whereas only 9·9 per cent of

FIG. 4.—Substandard housing areas. Districts given a high rate priority for clearance and renewal of the present housing. Oslo, Inner Zone.

TABLE I.—*Characteristics of Substandard Housing Areas Compared with "Inner Zone" of Oslo*

| | Substandard housing areas. | Inner Zone. | Substandard housing areas, % of Inner Zone |
|---|---|---|---|
| Population, 1936 | 36,350 | 275,150 | 13·2 |
| ,, , 1950 | 33,129 | 281,000 | 12·2 |
| Change in population, 1936/50 | −3,221 | +5,850 | |
| No. of rooms, 1948 | 21,854 | 221,271 | 9·9 |
| Persons per room | 1·52 | 1·25 | |
| No. of children 0–15 years | 5,240 | 45,950 | 11·4 |
| Children under public care | 138 | 860 | 16·1 |
| Youth registered for public care | 40 | 286 | 14·0 |
| Sentences 1949, drunkenness | 28 | 197 | 14·2 |
| ,, ,, , thefts etc. | 36 | 160 | 22·5 |
| Industry, units 1952* | 101 | 641 | 15·7 |

* Employing ten or more workers.

the residence rooms are found in these areas. Furthermore, 16·1 per cent of the children under public care are living in these districts whereas the proportion of all children under 15 years of age is 11·4 per cent. Of particular interest is the fact that as much as 22·5 per cent of all crimes for gain are committed by inhabitants of these " social problem-areas ". There has been a tendency to move out of these districts in later years.

Table II shows the mortality rate of gastric cancer in these areas, by age and sex, as compared with the rest of the Inner Zone and the Outer Zone respectively. The sub-standard housing areas shows an increased gastric cancer mortality in the three recorded age groups. The age- and sex-corrected mortality rate is 14·7 for these districts as compared with 10·0 for the rest of the Inner Zone. This difference is statistically significant ($X^2 = 15 \cdot 68$; df $= 6$; $0 \cdot 01 < P < 0 \cdot 05$).

TABLE II.—*Deaths from Gastric Cancer in Substandard Housing Areas Compared with Rest of Oslo*

| | | 50–59. | | 60–69. | | 70–79. | |
|---|---|---|---|---|---|---|---|
| | | M. | F. | M. | F. | M. | F. |
| Deaths from gastric cancer | Substandard housing areas | 7 | 7 | 24 | 9 | 18 | 15 |
| | Rest of Inner Zone | 52 | 34 | 79 | 55 | 78 | 89 |
| | Outer Zone | 20 | 13 | 35 | 15 | 50 | 32 |
| Population Dec. 1, 1950 | Substandard housing areas | 2,333 | 2,690 | 1,380 | 1,784 | 818 | 1,119 |
| | Rest of Inner Zone | 17,345 | 23,012 | 9,525 | 14,534 | 4,361 | 8,843 |
| | Outer Zone | 7,834 | 8,091 | 3,893 | 4,370 | 2,652 | 2,651 |
| Deaths per 10,000 per year | Substandard housing areas | 6·0 | 5·2 | 34·8 | 10·1 | 44·0 | 26·8 |
| | Rest of Inner Zone | 6·0 | 3·0 | 16·6 | 7·6 | 35·8 | 20·2 |
| | Outer Zone | 5·1 | 3·2 | 18·0 | 6·9 | 37·7 | 24·2 |

### DISCUSSION

The data presented in the cartographic analysis are generally in good conformity with the " concentric zone hypothesis " of Burgess (1925) according to which the most characteristic " slums " are to be found in areas surrounding business districts (Petersen, 1956). The inhabitants of these areas seem to be particularly exposed to the risk of developing gastric cancer.

On the other hand, certain objections may be mentioned.

First, the difficulties connected with the collection of relevant data are evident. Thus, it has been argued that some patients might have got into economical troubles and might possibly have moved into " slum " areas because of bad health such as gastro-intestinal disturbances which, in some cases, might have been gastric cancer or some possible precursor such as gastritis, gastric ulcer, polyps or pernicious anemia. This would imply that the low socio-economic status would be the effect rather than a (contributory) cause of the disease in some cases. The fact that most patients in our material have been living within the same areas for more than 15 years, however, may be a certain extent invalidate this argument. This and some other sources of error have been dealt with in our previous article (Torgersen and Petersen, 1956).

Second, the cartographic analysis apparently shows the limited value of using such crude correlates as " dwelling density ", " industry " etc., in such investigations. Thus, for instance, a most outstanding discrepancy is observed in certain districts with regard to dwelling density. Although a positive correlation between this factor and gastric cancer death rate seems to exist, some of the areas presenting a high dwelling density show a low rate of gastric cancer (some northern districts in Fig. 2, as compared with Fig. 1). Part of the explanation, however, may be that the high dwelling density in these districts is mainly due to the presence of many children which probably is a factor of negligible significance with regard to gastric cancer mortality. This point will be studied more closely in subsequent work.

The effect of socio-economic factors connected with residence in sub-standard housing areas (" slum clearance districts ") is indicated by the increased mortality rate of gastric cancer in these districts as compared with the rest of the Inner Zone.

It must be admitted, however, that the factor groups at present available for correlation with gastric cancer mortality are very crude. Thus, the transcription of " low socio-economic conditions ", " high dwelling density ", etc., into terms of etiology such as deficient nutrition or the ingestion of carcinogenic substances would seem a rather hazardous task at the present time. In further investigations, other factor groups of possible significance will be registered, and attempts will be made to transform some of these problems into the experimental field.

SUMMARY

An analysis has been made of the deaths from gastric cancer recorded on the death certificates of Oslo during the five-year period 1949–1953. Particular attention has been paid to various sources of error, as described in a previous article (Torgersen and Petersen, 1956).

Two types of investigation have been performed, namely cartographic analysis of small areas (" census tracts "), and comparisons between gastric cancer death rates of sub-standard housing areas (" slum clearance districts ") and the rest of the city.

The cartographic analysis outlines certain areas which show a particularly high death rate from gastric cancer (over 100 per 10,000 ; Fig. 1). Comparisons are made with location if industry, dwelling density, and sub-standard housing areas (Fig. 2–4).

The latter districts reveal a mortality rate from gastric cancer amounting to 14·7 as compared with 10·0 for the rest of the Inner Zone. This difference is significant statistically. A further characterization of these districts is given in Table I.

These investigations are consistent with the hypothesis that socio-economic factors are effective in the genesis of gastric cancer. The need for a more precise definition of such factors is emphasized.

We are indebted to Magister Egil Nilsen for statistical aid and to The Norwegian Cancer Society (Landsforeningen mot Kreft, Oslo) for economical support.

### REFERENCES

Burgess, E. W.—(1925) 'The Growth of the City : An Introduction to a Research Project.' Chicago.

Clemmesen, J. and Nielsen, A.—(1951) *Brit. J. Cancer*, **5**, 159.

COHART, E. M.—(1954) *Cancer*, **7**, 455.
GADE, F. G.—(1916) 'Undersökelser over Kraejtsygdommene i Norge.' *Videnskapsselskapets Skrifter I. Mat. naturv. klasse*, **7**, 76.
MACDONALD, I. AND KOTIN, P.—(1954) *Surg. Gynec. Obstet.* **98**, 148.
PATNO, M. E.—(1954) *Publ. Hlth Rep. Wash.*, **69**, 705.
PETERSEN, M.—(1955) 'Oslos geografiske struktur.' (Mimeographed, Oslo Town Planning Office.)
PETERSEN, M.—(1956) 'The Urban Fringe and Metropolitan Structure.' International Federation for Housing and Town Planning, Wien.
REGISTRAR-GENERAL—(1938) Decennial Supplement, England and Wales, Part IIa. 'Occupational Mortality, 1931.' London (H.M. Stationery Office).
RENNAES, S. AND ÖSTBERG, E. W.—(1955) *Brit. J. Cancer*, **9**, 7, 20.
STEINER, P. E.—(1952) *Cancer Res.*, **12**, 455.
STOCKS, P.—(1947) 'Regional and Local Differences in Cancer Death Rates.' London (H.M. Stationery Office).
TORGERSEN, O. AND PETERSEN, M.—(1956) *Brit. J. Cancer*, **10**, 292.